# Chimney Trial: study protocol for a randomized controlled trial

**DOI:** 10.1186/s13063-019-3764-y

**Published:** 2019-11-28

**Authors:** Elisa Mäkäräinen-Uhlbäck, Heikki Wiik, Jyrki Kössi, Maziar Nikberg, Pasi Ohtonen, Tero Rautio

**Affiliations:** 1 Oulu University Hospital, PL 21, 90029 OYS, Finland; 2 Päijät-Häme Central Hospital, Keskussairaalankatu 7, 15850 Lahti, Finland; 3 Västmanlands Hospital Västerås, 721 89 Västerås, Sweden

**Keywords:** Parastomal hernia prevention, Abdominoperineal resection, Rectal cancer treatment

## Abstract

**Background:**

Parastomal hernias (PSHs) are common, troubling the lives of people with permanent colostomy. In previous studies, retromuscular keyhole mesh placement has been the most-used technique for PSH prevention but results have been controversial. Additionally, surgical treatment of PSHs is associated with a high rate of complications and recurrences. Therefore, it is crucial to find the most effective way to prevent PSHs in the first place without an increased risk of complications. Due to a lack of adequate research, there is no clear evidence or recommendations on which mesh or technique is best to prevent PSHs.

**Methods/design:**

The Chimney Trial is a Nordic, prospective, randomized controlled, multicenter trial designed to compare the feasibility and the potential benefits of specifically designed, intra-abdominal onlay mesh (DynaMesh®-Parastomal, FEG Textiltechnik GmbH, Aachen, Germany) against controls with permanent colostomy without mesh.

The primary outcome of the Chimney Trial is the incidence of a PSH detected by a computerized tomography (CT) scan at 12-month follow-up. Secondary outcomes are the rate of clinically detected PSHs, surgical-site infection as defined by the Centers for Disease Control and Prevention (CDC), complications as defined by the Clavien-Dindo classification, the reoperation rate, operative time, length of stay, quality of life as measured by the RAND-36 survey and colostomy impact score, and both direct and indirect costs. For each group, 102 patients were enrolled at attending hospitals and randomized at a ratio of 1:1 by browser-based software to receive a preventive mesh or a conventional colostomy without a mesh. Patients will be followed for 1 month and at 1, 3, and 5 years after the operation for long-term results and complications.

**Discussion:**

The Chimney Trial aims to provide level-I evidence on PSH prevention.

**Trial registration:**

ClinicalTrials.gov, ID: NCT03799939. Registered on 10 January 2019

## Introduction

### Background and rationale

Abdominoperineal resection (APR) with permanent end-colostomy formation was introduced in the late twentieth century as a surgical method to treat distal rectal cancer in order to decrease the previously high incidence of local recurrence [1, 2]. Despite the rising trend of saving sphincter function, APR still remains the primary operation of choice for patients with low rectal cancer [3]. Even more, the low Hartmann’s procedure with permanent colostomy is increasingly performed in older and frailer patients who are not suitable for an anastomoses [4].

The reported incidence of parastomal hernias (PSHs) with permanent end colostomy rises to 81% after long-term follow-up [5]. Due to increased use of minimally invasive rectal cancer surgery and better survival, an increased incidence of PSHs might be expected [6, 7]. Many PSHs are asymptomatic, and clinical examinations can reveal only some of them compared with computed tomography (CT) scans [7, 8]. Most PSHs are diagnosed within 2 years after the construction of the stoma, but their incidence increases after longer follow-up [5, 9, 10].

The results of PSH repair are still unsatisfactory due to a high rate of complications [11]. Therefore, the initial focus should be on prevention, which is recommended in guidelines by the European Hernia Society [11].

Several randomized control trials (RCTs) and meta-analyses have shown positive results for synthetic meshes utilized as prophylaxis with various techniques and meshes [5, 6, 12–19]. Trials have demonstrated a lower incidence of PSHs without a higher risk of complications. However, despite the use of a prophylactic mesh, the incidence of PSHs has been surprisingly high. Clinically detected hernias are present in up to 10.6–16.4% of patients with a parastomal mesh, and the incidence of radiologically detected hernias is 32.4–36.6% on recent meta-analyses [18, 20]. Additionally, a recently published RCT [21] reported similar rates of radiologically detected PSHs at 1 year after open APR in patients with (32%) and without (34%) a prophylactic retromuscular sublay mesh.

Polyvinylidene difluoride mesh (PVDF, DynaMesh®-IPST, FEG Textiltechnik GmbH, Aachen, Germany) is a synthetic mesh with a 4-cm-long, central, seamless tube (chimney) designed to repair and prevent PSHs. There are three case series [22–24] published on its use as a prophylactic mesh. In 2008, Berger et al. [22] found no clinically detectable hernias by CT scan at 1-year follow-up. In another trial, [23] the incidence of PSHs was clinically found 3.2% and in CT scans 6.4% at the 1-year follow-up in a case series of 31 patients. The prophylactic method was safe with no unexpected complications. No RCTs have been published so far. The design of the mesh with a central tube forming a stocking-like lining around the bowel may be crucial for PSH prevention [22] and blocking stomal-orifice enlargement occurring with keyhole techniques [13].

### Objectives

The objective of this study is to compare prospectively in a randomized setting the feasibility and potential benefits of specifically designed, intra-abdominal, onlay mesh (DynaMesh®-IPST, FEG Textiltechnik GmbH, Aachen, Germany) with controls having conventional colostomy without mesh in patients operated on with minimally invasive surgery.

There are very few studies on specially designed PVDF mesh in PSH prophylaxis, but the concept of tight, tube-fashioned mesh surrounding the bowel and stomal orifice might be beneficial. We hypothesize that the high rate of PSHs seen in previous trials with retromuscular keyhole mesh is due to the central hole, and the drawback may be avoidable with the use of specially designed PVDF mesh.

### Trial design

The Chimney Trial is designed as a prospective, randomized, controlled, multicenter, single-blinded study of patients who had undergone either mini-invasive laparoscopic and robotic-assisted APR or low Hartmann’s procedure for rectal adenocarcinoma. The trial is independent from any kind of industrial sponsorship.

Previous research on PVDF mesh used for PSH prevention is scarce. We conduct and evaluate the complications and adverse events of PVDF mesh for safety reasons for 30 patients who have completed 30 days’ follow-up in a group with the mesh and another group with a conventional colostomy without a mesh. If there are 10% or more serious complications defined by Clavien-Dindo classification 3B in either group, the trial will be regarded as unethical and will be terminated. For the same safety reasons, there will be further analyses on the effectiveness and complications when 30 patients in both groups have reached the 1-year follow-up. If the PSH rate is increased by more than 35% in the control group compared with the PVDF-mesh group or there are 10% or more complications defined by Clavien-Dindo classification 3B in either group compared with the other group, the trial will be terminated as unethical to continue. In case of terminating the trial prematurely, the data collected and potential complications are published.

## Methods/design

### Study setting

This study is a multicenter study involving several Nordic hospitals. Hospitals currently participating in this study are Oulu University Hospital, Helsinki University Hospital, Turku University Hospital, Tampere University Hospital, Jyväskylä Central Hospital, and Seinäjoki Central Hospital in Finland and Västmanlands Hospital Västerås in Sweden. It is expected that hospitals from other Nordic countries, such as Norway and Denmark, will join the trial later.

Oulu University Hospital and Seinäjoki Central Hospital began recruitment in February 2019 with other hospitals soon to following.

### Eligibility criteria

**Fig. 1:**
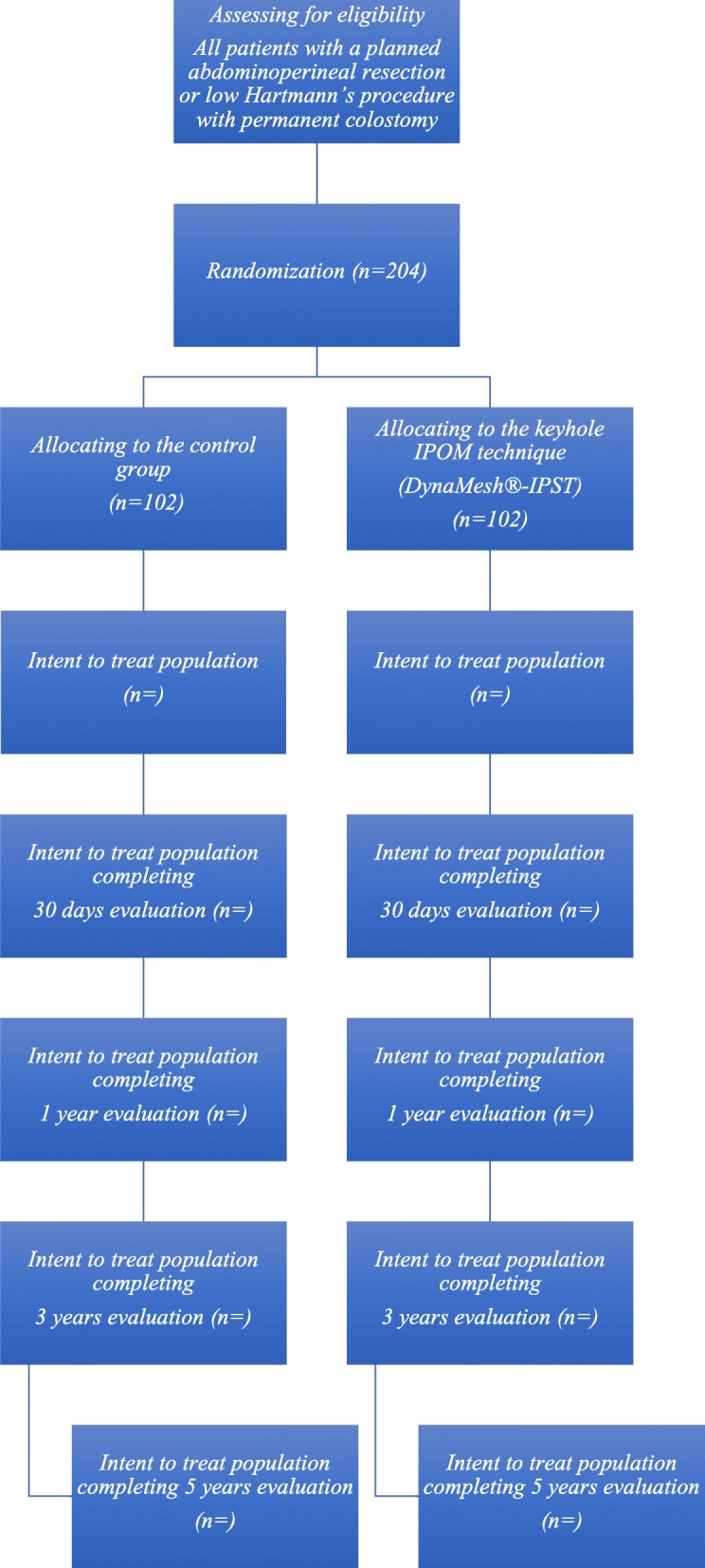
Flow chart

All patients who fulfill the inclusion criteria without meeting any of the exclusion criteria are considered to participate in the trial at any of the hospitals during the study period (Fig. 1). Patients are enrolled in the study in the outpatient department at a visit prior to surgery. All patients who undergo APR or Hartmann’s procedure for rectal adenocarcinoma during the study period in each attending hospital are recorded without identification details for later analysis of selection biases.

If a patient refuses to attend the trial, they are treated according to routine practice.

### Inclusion criteria

- APR or low Hartmann’s procedure for rectal cancer with curative intent and permanent end colostomy, either by laparoscopic technique or robotic-assisted APR
- 18 years of age or older
- Patient has a life expectancy of at least 12 months
- Patient signs the informed consent and agrees to attend all study visits

### Exclusion criteria

- APR or Hartmann’s resection by laparotomy or conversion to laparotomy
- Complications requiring laparotomy during postoperative treatment on the surgical ward
- Patient with a comorbid illness or condition that would preclude surgical treatment (American Society of Anesthesiologists (ASA) 4–5)
- Patients with concurrent or previous malignant tumors within 5 years before study enrollment
- Patients with grade-T4b tumors that impose a multi-organ resection
- Rectal malignancy other than adenocarcinoma
- Patients undergoing emergency procedures
- Planned rectal surgery along with major concomitant procedures (e.g., hepatectomies, other intestinal resections)
- Metastatic disease with no possibility of curative surgery
- Pregnancy or suspected pregnancy
- Patients living geographically distant and/or unwilling to return for follow-ups or comply with all study requirements
- Active abdominal infection at the time of surgery
- Previous surgery at the colostomy site
- Language barrier or other reasons why informed consent is not possible

### Interventions

Perioperative care includes the assessment and optimization of medical risk factors, thromboprophylaxis with low-molecular-weight heparin and elastic antiembolic stockings, standard anesthesia and the avoidance of hypothermia. Antibiotic prophylaxis and mechanical bowel preparation are accomplished according to the hospital protocol. Postoperative treatment on the surgical ward is accomplished according to standard enhanced recovery after surgery (ERAS) protocols.

### Surgical technique

**Fig. 2:**
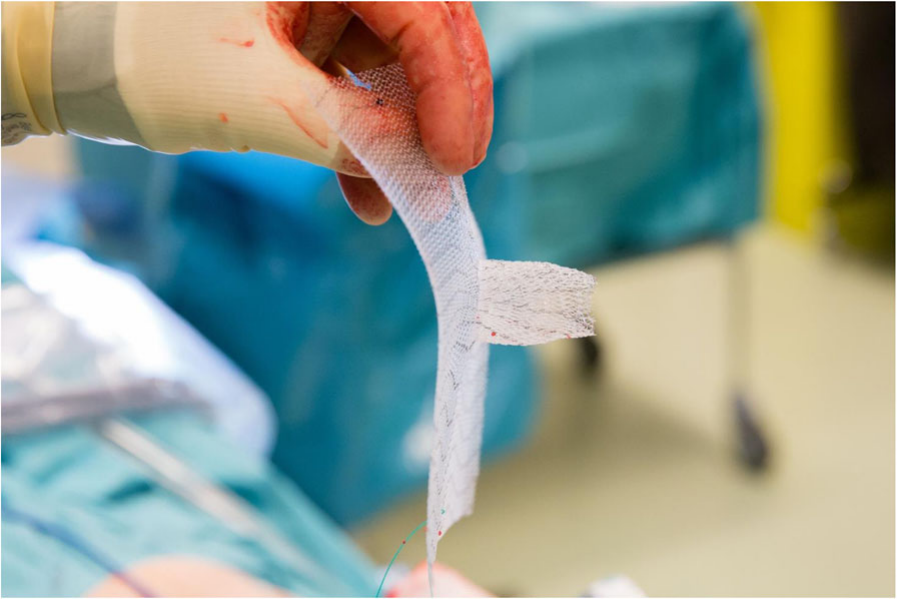
The polyvinylidene difluoride (PVDF) mesh (DynaMesh®-IPST, FEG Textiltechnik GmbH, Aachen, Germany) with the 4-cm long funnel

**Fig. 3:**
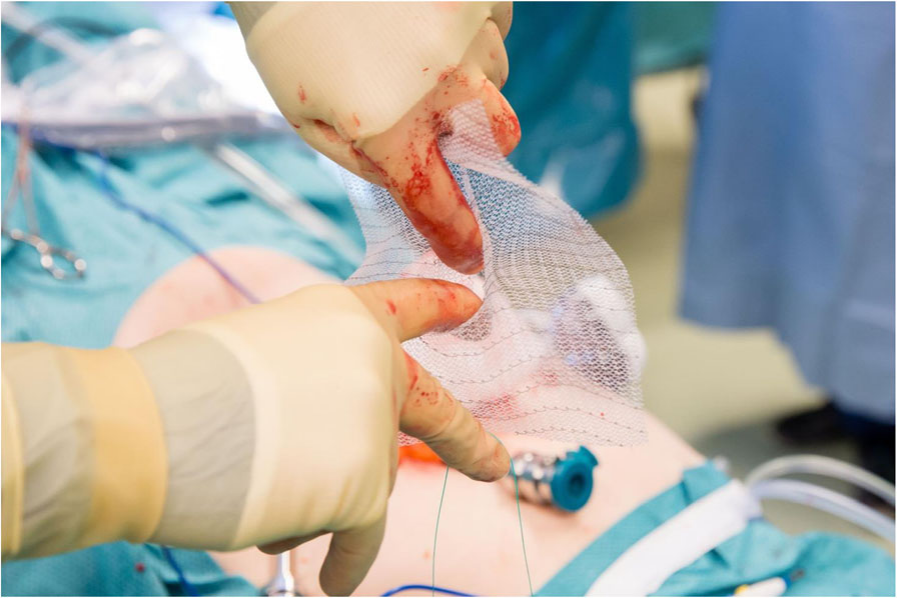
The funnel-shaped tube is stretched with fingers to match the diameter of the bowel

**Fig. 4:**
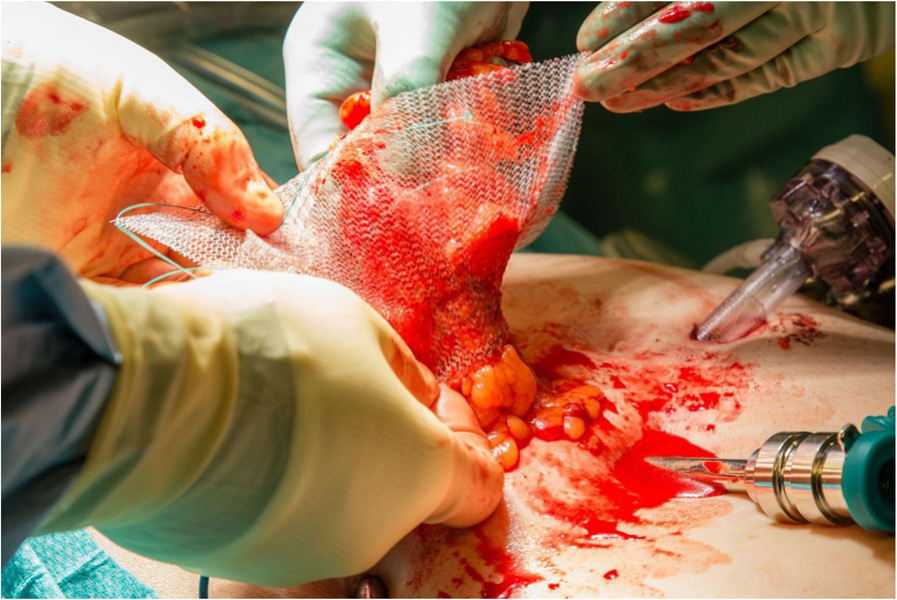
The bowel is brought through the funnel oriented dorsally

**Fig. 5:**
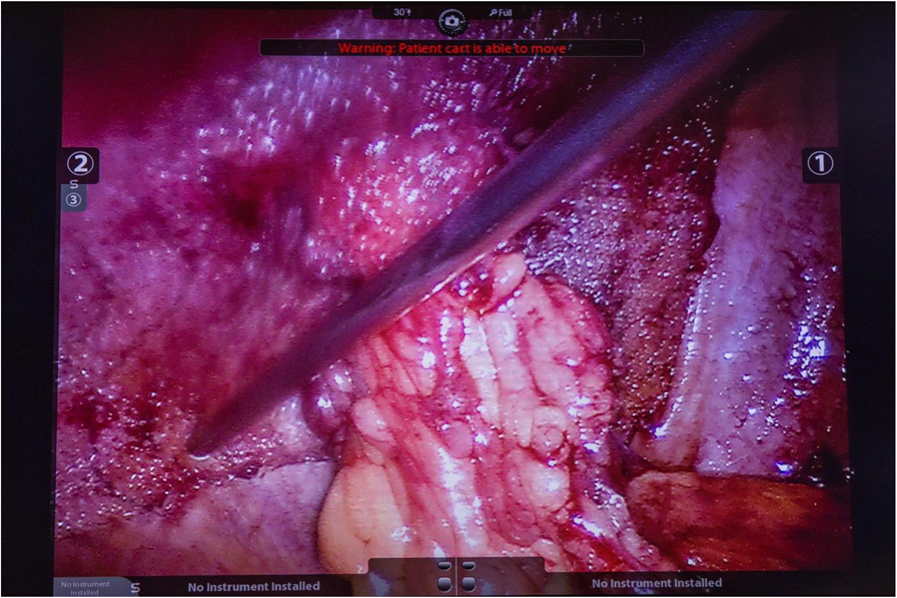
The polyvinylidene difluoride (PVDF) mesh is attached to the abdominal wall using absorbable tackers

PVDF mesh (DynaMesh®-IPST, FEG Textiltechnik GmbH, Aachen, Germany) is placed on the intraperitoneal surface as described by Berger et al. [22], Conde-Muino et al. [23] and Köhler et al. [24]. The bowel forming the colostomy is closed with a linear-stapling device. The trephine is created by excision of the skin at the site previously marked by a trained ostomy nurse. Subcutaneous tissue is not excised. A cross-shaped incision is made to the anterior rectus sheath. The rectus abdominis muscle is split in the direction of the fibers, and the posterior rectus sheath is opened longitudinally. A 15 × 15-cm mesh with a tube length of 4 cm and a width of 2 cm is used (Fig. 2). The tube is stretched using the surgeon’s fingers to match the diameter of the bowel (Fig. 3). The bowel is brought through the orifice and then through the saline lubricated tube in the PVDF mesh (Fig. 4). The mesh is translocated into the intra-abdominal space with the funnel oriented dorsally and fixed in the intraperitoneal onlay position by absorbable tackers (Securestrap™, Ethicon) using the double-crown technique as described and pictured previously by Köhler et al. [24] (Fig. 5) The corners of the mesh are fixed first, then the tackers are attached every 2 cm on the outer row. The inner row is fixed at 12, 3, 6, and 9 o’clock positions. The stoma is fixed and everted with absorbable monofilament sutures to the skin just above the skin level. A catalog of the operative technique will be sent to all participating surgeons to standardize the procedure.

In the control group, the colostomy is formed by the identical method as described above. The only difference is that there will be no mesh.

### Outcomes

The primary endpoint of this study is the incidence of PSHs, either symptomatic or asymptomatic, detected by a CT scan during the 12 months’ post-surgery follow-up. A CT scan with the Valsalva maneuver being performed is performed at the 1-year and 3-year follow-up after rectal adenocarcinoma operation as a part of routine follow-up protocol and to detect the incidence of radiological PSHs. All patients are also assessed at each follow-up point (Table 1) by an experienced surgeon to detect clinical PSH or complication of stoma as secondary outcome. There will be clinical evaluation without CT scan to detect any clinical PSH as long-term follow-up at 5 years after surgery.

**Table 1:** Participant timeline

Schedule of events	Baseline	Procedure	Discharge	30 days ±3 days	1 year ±14 days	3 years ±30 days	5 years ±30 days	Unscheduled visit
Informed consent	X							
Demographics and medical history	X							
QoL (RAND-36)	X			X	X	X	X	
QoL (colostomy impact score)				X	X	X	X	
Procedure details		X						
CT scan findings					X	X		
Protocol deviation	X^a^	X^a^	X^a^	X^a^	X^a^	X^a^	X^a^	X^a^
Complications		X^a^	X^a^	X^a^	X^a^	X^a^	X^a^	X^a^
Study closure form							X^b^	

Legend: *CT* computed tomography, *QoL* quality of life

^a^Complete if applicable

^b^Complete when lost to follow-up, consent withdrawal or subject has completed all study-related visits

All CT scans are analyzed by two independent radiologists. Measurements are recorded of fascial defect at stoma, the size of any possible PSH sac and fascial defect of a hernia, the content of the hernial sac, the location of the stoma and other hernias. The hernias will be graded according to the European Hernia Society criteria [25].

Primary outcome

- PSH, either symptomatic or asymptomatic, detected by CT scan during 12 months’ follow-up

Secondary outcomes

- Incidence of CT-detected PSH during 3-year follow-up
- Incidence of clinically detected PSH during 1-, 3-, and 5-year follow-ups
- PSH-operation-free survival at 3 and 5 years
- Surgical-site infection (SSI) rate
- Clavien-Dindo grade I–V complications at 30 days postoperatively
- Stoma-related complications and problems during long-term follow-up
- Stoma-related readmissions
- Reoperation rate
- Operative time
- Length of stay (LOS) in days
- Quality of life (RAND-36, colostomy impact score)
- Medico-economic sub-study including direct costs at hospital and indirect costs caused by sick leave
- Radiological sub-study including definition of abdominal wall measurements and location of stoma

### Radiological sub-study

- Subcutaneous abdominal fat in centimeters on the contra-lateral side of the stoma
- Distance of the medial part of the stoma to midline (umbilicus as the midline definition)
- Area of the stoma aperture (2 cm width × height/2)

The SSI is defined and recorded per the Centers for Disease Control and Prevention (CDC) SSI definition [25].

Clavien-Dindo classification is utilized for complications. All related costs are analyzed in detail. The direct costs, including the meshes, resources and stay at the hospital, are monitored, and the indirect costs from losses of productivity are recorded.

### Pre-intervention data

- Age
- Sex
- ASA class
- Weight, height
- Other illnesses and medications
- Smoking history
- Previous hernias, either symptomatic or asymptomatic
- Neoadjuvant treatment
- Preoperative hemoglobin and carcinoembryonic antigen (CEA)
- Quality of life defined by the RAND-36
- Informed consent and patient information
- Randomization

### Intervention data

- Antibiotic prophylaxis
- Operating time
- Resources used during the operation
- Total blood loss

### Post-intervention data

- Length of postoperative ileus (POI) measured in days and defined by air in the stoma
- Re-operation rate
- Complications as defined by Clavien-Dindo classification
- Incidence of SSI as defined by the CDC
- Incidence of either clinically or radiologically detected PSHs
- Quality of life at each follow-up
- Problems and complications with stoma
- Tumor–nodes–metastases (TNM) score
- Hemoglobin and CEA at each control
- Oncological adjuvant treatment given

All exceptions to the protocol are recorded and explained in detail at each time point.

### Sample size

To calculate a sample size needed to compare the two groups, we estimated a 6.4% rate of PSH and 34% PSH on a CT scan for the PVDF-mesh group and control group during 12 months’ follow-up [21, 23]. Assuming α = 0.05 and power = 90%, we would need 51 patients per group. Furthermore, assuming a 5-year drop-out rate of 50%, 102 patients per group are needed to reach statistically significant results also during long-term follow-up.

All analyses will be performed by, or under the guidance of, professional statisticians and following the Consolidated Standards of Reporting Trials (CONSORT) guidelines [26].

### Recruitment

All patients who will undergo laparoscopic or robotic-assisted APR or low Hartmann’s procedure for rectal adenocarcinoma at each study site are considered for the trial at the time of their visit to the outpatient department prior to surgery. After receiving the proper information on the possible advantages and disadvantages of the intervention, and voluntarily signing an informed consent form, the subject is enrolled in the Chimney Trial. Participating investigators are qualified colorectal or general surgeons experienced in the surgical management of patients with colorectal adenocarcinoma and either laparoscopic or robotic-assisted APR or low Hartmann’s procedure. Each hospital’s contribution to the study is limited to no less than 20 cases. The enrollment will last for approximately 2 to 3 years in attending hospitals.

## Methods

### Allocation

All analyses will be performed by or under the guidance of professional statisticians and following the CONSORT guidelines [26].

Patients are randomly allocated to the study group according to a computer-generated list, compiled by a biostatistician who is not involved in the clinical care of trial patients. The randomization is performed in blocks, where the block size varies randomly between two, four, and six patients. A separate randomization list is created for each center. The software designed for the study is used to randomize the patients. After confirmation of patients’ eligibility and their willingness to participate, the randomization is completed at the outpatient visit prior to surgery and the patients are kept blinded to study technique.

### Blinding

Patients are blinded to the randomization group during their primary stay at the hospital. For safety reasons, their group designation is stated in the patients’ medical files for direct access in case of complications. The patient has direct access to their medical records after hospitalization and, therefore, the blinding is not possible to maintain. Patients are assigned at the control visits by a surgeon not involved in the study and without accessing the randomization group. The independent radiologists analyze the CT scan without access to the randomization group information.

### Data collection, management and analysis

A dedicated electronic database and randomization software program is used to host the clinical trial data for this study. All electronic case report forms (eCRFs) are handled with a special trial ID. Access to the database is limited to the main investigators. All data requested on the eCRFs will be recorded. All missing data will be explained.

Data collection is the responsibility of the principal investigator at each study site and is reviewed by the study group.

Reasons for withdrawal will be documented carefully. The investigator will attempt to contact the subjects at least three times prior to designating them as lost to follow-up. The investigator will document the date and type of attempted communication. If a subject cannot be reached during the visit window, a missed visit is recorded; after three consecutive missed visits, a subject is considered lost to follow-up and a study exit form will be completed on the electronic database. Any data on a subject’s participation and procedures until the withdrawal will be analyzed within the research.

All complications are recorded and monitored using a specific eCRF. Data of all complications is later published as part of the trial results.

### Statistical methods

The primary endpoint will be the incidence of PSHs detected by CT scan with 95% confidence intervals for all groups at 1-year follow-up. Secondary outcomes are the incidence of PSHs at 3- and 5-year follow-ups and the development of quality of life during the follow-ups. The analyses will be based on the intention-to-treat principle. The primary endpoint and other categorical data will be analyzed by the χ^2^ test or Fisher’s exact test. Analysis of variance (ANOVA) will be used for continuous variables. For repeatedly measured data, the linear mixed model (LMM) will be used for continuous data and the generalized linear mixed model (GLMM) will be used for categorical data. Multiple imputations of missing outcome data will be used for sensitivity analyses. The SPSS statistical programs (IBM Corp. 2016. IBM SPSS Statistics for Windows, Version 24.0. Armonk, NY, USA) and SAS (version 9.4, SAS Institute Inc., Cary, NC, USA) will be used for the analyses.

## Ethics and dissemination

### Research ethics approval

This study follows the Declaration of Helsinki on medical protocol and ethics. Each participating hospital applies for study permission at their unit. Central ethical approval has been confirmed from the Ethical Committee at Oulu University Hospital (ref approval no. 324/2018) and we will not begin recruiting at other centers in the trial until local ethical approval has been obtained.

### Protocol amendments

Important protocol modifications are communicated with the Oulu University Hospital Ethics Committee by amendments. All modifications are also registered at ClinicalTrials.gov.

### Confidentiality

Patient confidentiality will be strictly maintained. Patients will be assigned a study ID, and all data will be handled without name or personal Social Security number. Access to patient records is limited to the study group and the investigator-delegated study coordinator.

## Discussion

The aim of this study is to assess, in a randomized, multicenter setting, the safety and efficiency of specially designed, funnel-shaped PVDF mesh (DynaMesh®-IPST, FEG Textiltechnik GmbH, Aachen, Germany) in PSH prevention compared with a control group without a mesh, all being operated on using mini-invasive laparoscopic/robotic methods. The hypothesis is that the high incidence of up to 32% of PSHs detected on CT scans after retromuscular sublay mesh [21] can be reduced and further repair of PSHs can be prevented with the use of a chimney-like tube in the PVDF mesh.

Research on specially designed PVDF mesh as prophylaxis is limited but promising with a PSH incidence rate of 6.4% at 1-year-CT follow-up [23]. No RCTs using this mesh exist so far. All previous case series [22–24] included a small number of patients with no controls thus providing inadequate evidence for efficiency and safety for more routine use of this prophylactic mesh.

The focus of the current study is on the incidence of PSHs at both short- and long-term follow-up.

PSHs are not only graded by European Hernia Society Classification [25], but also the exact size and content of hernias upon the Valsalva maneuver are measured as well as the development of measurements throughout the follow-up. Radiological risk factors for PSH development will be determined as part of radiological follow-up. All symptoms caused by a stoma itself or a PSH are recorded to analyze the clinical significance of clinically or radiologically detected PSHs. The quality of life is measured by both RAND-36 and the colostomy impact score and recorded throughout the follow-up period as part of defining the clinical significance of PSHs. The re-operation rate and operations done for PSH are recorded in both groups.

This trial is designed as a single-blinded study for safety reasons. In the case of serious complications demanding re-operation, it is crucial for decision-making purposes to always have instant access to all technical aspects of the surgery. Patients are blinded to the method used during their hospital stay. Unfortunately, it is impossible to blind patients beyond that time due to direct access to the national medical database including all medical records and hospital stays.

As current results from the use of the most evaluated keyhole technique in PSH prevention are unsatisfactory, more trials are needed to define the efficiency and safety of other methods to prevent PSH. As previous research on the PVDF mesh used in this trial is limited, there are clearly predefined safety limits of PSH incidence and complications to determine when to prematurely finish the trial as unethical to continue. For the same reason, the trial is designed to compare the mesh group to control group without any mesh to detect the objective efficiency and safety of funnel-shaped PVDF mesh. Since there is a lack of long-term results of PSH prevention, the sample size is estimated to reach statistically significant results at the long-term follow-up at 5 years.

## Conclusions

The Chimney Trial aims to provide level-I evidence on PSH prevention. The trial considers the economic aspects, effectiveness and safety profile with the scarcely trialed PVDF mesh at both short- and long-term follow-up.

## Trial status

The trial started recruiting on 5 February 2019. The recruitment is estimated to be complete by the end of 2021. The protocol date of protocol version 1 is 20 November 2018.

## Data Availability

The datasets generated and/or analyzed during the current study are not publicly available due to Finnish laws on privacy protection but are available from the corresponding author on reasonable request.

## Abbreviations

APR: Abdominoperineal resection
CDC: Centers for Disease Control and Prevention
CEA: Carcinoembryonic antigen
CRF: Case report form
CT: Computed tomography
ERAS: Enhanced recovery after surgery
GCP: Good Clinical Practice
LoS: Length of stay
PSH: Parastomal hernia
PVDF: Polyvinylidene difluoride
SSI: Surgical-site infection
TNM: Tumor–nodes–metastases
VAS: Visual analog scale
